# Quality of Fish-Oil-Based Dietary Supplements Available on the Italian Market: A Preliminary Study

**DOI:** 10.3390/molecules26165015

**Published:** 2021-08-19

**Authors:** Teresina Nevigato, Maurizio Masci, Roberto Caproni

**Affiliations:** Council for Agricultural Research and Economics (CREA), Research Centre for Food and Nutrition, 00178 Rome, Italy; maurizio.masci@crea.gov.it (M.M.); roberto.caproni@crea.gov.it (R.C.)

**Keywords:** dietary supplement, fish oil, quality control, AOAC method, fatty acids, omega-3, gas chromatography-mass spectrometry

## Abstract

The global market of food supplements is growing rapidly with a large turnover. Fish oil supplements represent a significant part of this turnover as they are believed to have important health benefits. Conversely, there are few papers in the literature about the quality control of fish oil capsules. As prior studies illustrate, a perfect agreement with the label is rarely found, and in some isolated cases, large amounts of soybean oil are also detected, indicating a true adulteration rather than a non-compliance with the label. None of the available studies refer to the Italian market, which ranks first in Europe in the consumption of food supplements. In this present communication, a quality control of fish-oil-based supplements from the Italian market was carried out for the first time. With minor deviations, all results showed substantial agreement with the label. However, the most important conclusion from this research is that compliance with the label is not enough to judge a product of good quality. The analysis of the overall fatty acid composition showed that some supplements have a high level of saturated fatty acids, and therefore they did not undergo a proper purification process. This may represent a safety issue since the purification process also allows the removal of toxic contaminants.

## 1. Introduction

It is widely recognized that fish consumption prevents cardiovascular diseases and acts positively against other important pathologies. This is due to the composition of fatty acids rich in omega-3, also known as n-3 fatty acids or n-3 long-chain polyunsaturated fatty acids (n-3 LC-PUFA), with eicosapentaenoic (EPA) and docosahexaenoic acid (DHA) as the major representatives. The amount of EPA + DHA consumed in a diet is one of the most important parameters in preventing coronary heart disease [1].

Fish-oil-based food supplements have had a growing market in recent years because they are expected to have the same beneficial effects as fish consumption. The global omega-3 supplement market size was valued at USD 5.18 billion in 2019, and is expected to expand at a compound annual growth rate (CAGR) of 8.4% from 2020 to 2027 [2]. Italy is the leading consumer of food supplements in Europe with a market share of 23% and a turnover of EUR 3.3 billion in 2018 [3]. Regarding fish oil supplements, in 2016, four million packs of omega-3 supplements were purchased in Italy for a total cost of EUR 84 million [4].

Despite this high turnover, scientific works aimed at controlling the quality of these products are scarce, and data exist for only six countries in the world: Australia [5], Brazil [6], Finland [7], New Zealand [8], Poland [9,10], and the USA [11,12,13,14,15], which are where the composition of fish oil supplements has been the most extensively investigated. Kleiner et al. analyzed 47 commercial omega-3 supplements and found that over 70% of the samples tested did not contain the stated label amount of EPA or DHA [13], similar to what was measured by Ritter et al., where half of the supplements did not meet the label claims [12]. Kowalski et al. observed that manufacturers declare higher levels of fatty acid content compared to the results obtained during the analysis [10], while in the study by Galuch et al., two brands were discovered with the addition of large amounts of soybean oil, leading the final consumers to ingest this low-cost oil believing that they were consuming adequate doses of EPA and DHA [6]. Often, when the product does not comply with the label, it has a measured content that is 80–90% of what is declared by the producer.

EPA and DHA are not the only indicators of quality. Chee et al. analyzed all fatty acids in eight commercially available fish oil capsules and found an average amount of EPA and DHA essentially in compliance with the label, but they concluded by saying: “These capsules contain as much saturated fat as they contain omega-3 fatty acids” [11]. It must be borne in mind that producing a good omega-3 supplement involves accurate purification steps through which free fatty acids, heavy metals, colored compounds, and other contaminants are removed from raw fish oil. Generally, the short-path distillation technique is used [16], also named molecular distillation, a special type of very-high-vacuum distillation. An additional process involves the concentration of fish oils, which leads to a higher total omega-3 content and a higher concentration of EPA and DHA together with the removal of saturated fatty acids. This step is frequently accomplished by winterization [17]. Such processes are potentially expensive, and some companies may choose not to carry them out; however, they may still be in compliance with what is declared on the label for EPA and DHA. However, the health impact may be different with respect to a purified product. Mason et al. analyzed three top-selling fish oil supplements in the USA and, from the measured fatty acid composition, concluded that the observed level of saturated fat may interfere with the intended/potential biological benefits of the studied products [15].

The practice of not purifying the product may have negative effects on safety since the lack of purification does not only result in keeping the saturated FAs at a high level. An important consequence of not purifying fish oil is to leave potential quantities of toxic contaminants such as POPs and heavy metals, for example, which can be present in fish oils.

In the present work, three popular brands of fish oil supplements available on the Italian market were analyzed. To this purpose, a variation of the AOAC Official Method 991.39 for Fatty Acids in Encapsulated Fish Oils [18] was applied.

It is the first time that the Italian market of fish oil supplements has been investigated, and the research is relevant since the product quality of fish oil supplements in Italy was unknown until now.

## 2. Results and Discussion

Table 1 shows the fatty acid (FA) composition of the three supplements analyzed. The values in the last row (total FAs) were experimentally obtained by the Kinsella method (Section 3).

A total of 46 FAs were detected in supplement no. 1, while in supplements no. 2 and no. 3, 55 and 47 FAs were detected, respectively.

The 27 main fatty acids listed in Table 1 represent most of the total, 85% in supplement no. 1, 86% in supplement no. 2, and 91% in supplement no. 3.

**Figure 1:**
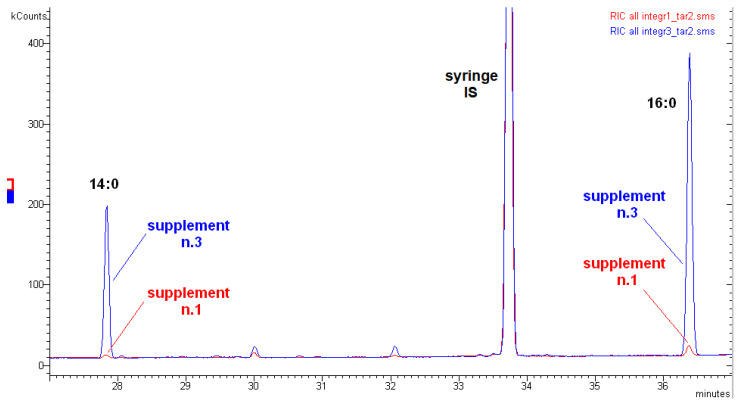
GC-MS chromatograms of supplement no. 1 and supplement no. 3 in full scan mode, time range 27–37 min. Chromatograms are overlaid. “Syringe IS” was added to the vials just before GC-MS injections to check the run-to-run repeatability of the retention times. As “syringe IS”, methylated C15:1 n-5 fatty acid was used.

It is immediately evident that the supplement no. 3 is of lower quality than supplements nos. 1 and 2 by simply observing the Σ saturated fatty acids (ΣSFA) value. The series ΣSFA-ΣMUFA-ΣPUFA as mg/100 mg oil is equal to 4.54-11.36-60.66 (5-11-61) for supplement no. 1, 7.42-12.82-55.42 (7-13-55) for supplement no. 2, and 24.67-20.96-38.08 (25-21-38) for supplement no. 3. This indicates that an effective purification step was carried out for supplements nos. 1 and 2 to eliminate the less valuable saturated fatty acids; the values for nos. 1 and 2 are not typical of fish oil, while the values for supplement no. 3 are [19]. It should be observed that the value of 16:0 (palmitic acid) is generally one of the most concentrated fatty acids in fish oils. In supplements no. 1 and no. 2, palmitic acid is instead one of the less concentrated FAs (0.16 and 1.32 mg/100 mg oil, respectively), while in the supplement no. 3, it is one of the most concentrated (13.80 mg/100 mg oil). A similar situation can be observed for 14:0 (myristic acid). Furthermore, the two saturated FAs, 12:0 and 13:0, are not detectable in supplement no. 1 and no. 2. On the contrary, they are present in supplement no. 3, even if in low quantity (the same low quantity generally observed in fish oils). Figure 1 shows the GC-MS chromatograms of supplements no. 1 and no. 3 in comparison, in the time range of 27–37 min.

A good purification process was observed for supplement no. 1, where two of the most concentrated saturated FAs in fish oils, 14:0 and 16:0, were almost completely eliminated from the sample, unlike supplement no. 3, where they were practically at the original average level found in fish oils.

In addition to the significant presence of saturated fatty acids, supplement no. 3 had a total omega-3 content of only 35 mg/100 mg oil compared to 57 and 52 mg/100 mg oil in supplements no. 1 and no. 2, respectively. For EPA and DHA, we observed a noticeable difference between supplement no. 3 and the other two. The measured EPA content in no. 3 was half that of no. 1 and two-thirds that of no. 2, while the measured DHA content in no. 3 was about two-thirds that of no. 1 and no. 2. In terms of percentages, the sum of EPA plus DHA represented 58% and 52% of total fatty acids in supplements nos. 1 and 2, respectively, while in supplement no. 3, it was 32%. This would force the consumer to take more oil from supplement no. 3 to obtain the same quantity of bioactive molecules with possible side effects, such as the intake of contaminants in non-negligible quantities, if the product was not perfectly purified.

Compliance with the label is shown in Table 2. In supplements no. 1 and no. 2, EPA is about 80% of the amount declared, while DHA is 91–98%. This is a frequent observation. In their study of marine oil capsules, Chee et al. measured 82.4% of labeled content regarding EPA and 90% regarding DHA [11]. Similar findings have been reported in other studies [10,12,13]. In a study of omega-3 fatty acid dietary supplements by the U.S. Department of Agriculture, Dietary Supplement Ingredient Database Team, a statistically significant difference between the measured and label level of EPA was observed in that the measured content was lower than the declared one [20]. Supplement no. 3 slightly exceeds the label statement for both EPA and DHA, but this does not necessarily mean higher quality, as explained above. Rather, in some cases, ingredients may be added by producers in amounts exceeding the label claims in order to compensate for losses during shelf life [20].

By considering the 27 main fatty acids, the ratio Σn-6/Σn-3 is 0.06, 0.06, and 0.05 for supplement no. 1, no. 2, and no. 3, respectively (Table 1). These values are well in compliance with the current guidelines, which recommend not to exceed the value of 1 in the diet for the ratio Σn-6/Σn-3. Such values were to be expected for products derived from fish oil, which are also used to rebalance the diet. 

## 3. Materials and Methods

A variation of the AOAC Official Method 991.39 for Fatty Acids in Encapsulated Fish Oils was applied. The AOAC Method 991.39 determines the area percentages of 24 fatty acids and the absolute weights (mg/g sample) of EPA and DHA only. The variation used in the present work provides the absolute weights of 27 fatty acids, including EPA and DHA. It was published and validated in 2012 [19].

To ensure that the procedure used here provides accurate and reliable results, the fish oil test material developed by fapas^®^ (The Food and Environment Research Agency, Sand Hutton, York, UK) was analyzed. In the test material specification sheet, the assigned values and the satisfactory ranges were reported. As shown in Table 3, the measured values are in good agreement with the declared ones, demonstrating that the entire procedure applied in our laboratory, from sample preparation to instrumental determination by GC-MS, is accurate.

### 3.1. Reagents and Derivatization

Three popular brands of fish oil-based supplements were purchased from retailers in Italy. All products are registered with the Italian Ministry of Health according to the law. Four capsules for each brand were opened, and approximately 10 mg of the pooled oil were methyl derivatized. Derivatization was carried out with BF_3_ methanol solution (AOAC Method 991.39), followed by extraction with n-hexane, as already reported [19]. Individual analytical standards of fatty acids were purchased as methyl esters either from Merck KGaA^®^ or from Larodan^®^ (Solna, Sweden). After the purchase, the pure FA standards were dissolved in n-hexane and stored at a temperature of −30 °C by using the Certan^®^ capillary bottles, i.e., vials specially designed for optimum storage available from Merck KGaA^®^, Darmstadt, Germany.

### 3.2. Saponification

An aliquot of oil from each brand was used to derive the total quantity of fatty acids per 100 mg of oil. The Kinsella method was used to this aim [21]. Briefly, about 100 mg of oil was subjected to saponification using 10% alcoholic KOH, the nonsaponifiable material was extracted with n-hexane, and the residual soaps were acidified to pH 1.5. The free fatty acids were extracted with n-hexane, dried in a tared vial, and the weight of fatty acids was determined. The total FAs as mg/100 mg oil for each one of the three supplements are shown in Table 1. As reported by Kinsella, “these data are used to calculate the weights of individual fatty acids separated by gas chromatography”.

### 3.3. Instrumental Analysis

The instrument used was a Varian 3900 gas chromatograph connected to a Saturn 2100T mass spectrometer (GC-MS) equipped with an ion trap analyzer (Varian^®^, Palo Alto, CA, USA). Injections were made in split mode (15:1) with an injection volume of 1 µL. Injections showed excellent retention time repeatability as evidenced by the syringe internal standard used (Figure 1). The capillary column installed was a CP-WAX 52 CB (60 m × 0.32 mm I.D., 0.50 µm film thickness) from Chrompack^®^, Middelburg, the Netherlands, which is equivalent to the Carbowax-20M column (AOAC 991.39). Mass spectra were obtained in electron ionization (EI) mode at 70 eV. Ion trap temperature was 180 °C. The selected mass to charge ratio to acquire was in the 40–440 *m/z* range. Analyses were carried out in full scan mode. The limit of detection was 0.005 mg FA/100 mg oil.

**Figure 2:**
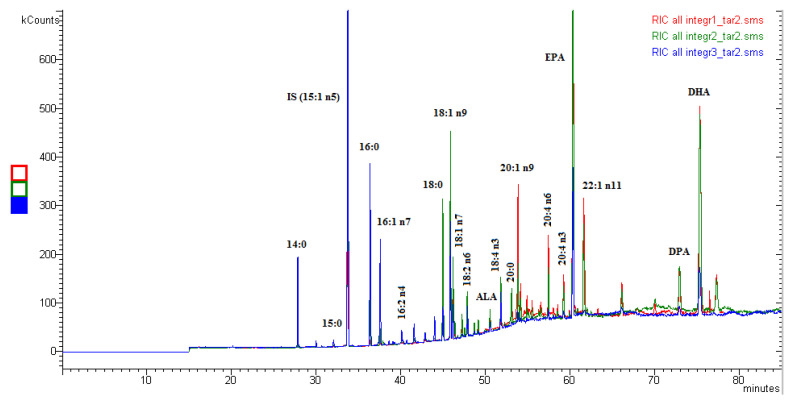
GC-MS chromatograms of the three supplements analyzed. Chromatograms are overlaid.

**Figure 3:**
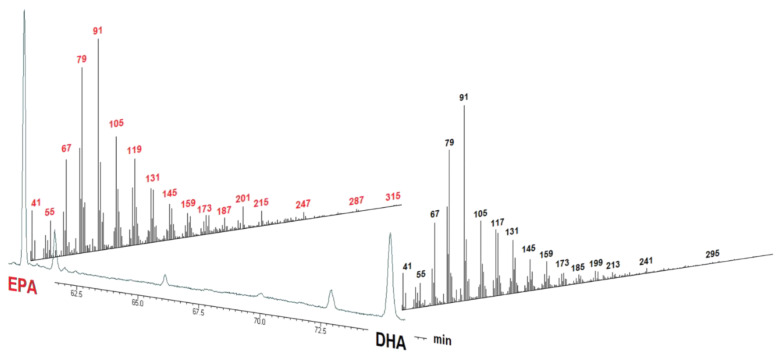
Supplement no. 2, GC-MS chromatogram in the time range 60–76 min, together with the mass spectra of EPA and DHA displayed in the third dimension.

GC-MS chromatograms were accurately reprocessed by performing a peak-by-peak identification. All FA peaks present in the sample until the signal-to-noise ratio of 3 were integrated. Detector correction factors for EPA and DHA (AOAC 991.39) were experimentally calculated ex novo [22] and applied. Figure 2 shows the GC-MS full scan chromatograms of the dietary supplements analyzed, while in Figure 3, the GC-MS chromatogram of supplement no. 2 in the time range 60–76 min is displayed together with the mass spectra of EPA and DHA.

## 4. Conclusions

Three fish oil supplements from popular brands in the Italian market were analyzed. Apart from a slight difference between the measured and declared content of EPA (frequently reported in the literature), supplements nos. 1 and 2 have a higher quality than supplement no. 3, the cheapest one. This last has a higher content of saturated fatty acids, thus suggesting that it has undergone incomplete or no purification. Furthermore, the lower content of omega-3 in supplement no. 3 may force the consumer to take more oil, with possible side effects. The lack of the purification process prevents the contaminants originally present in the fish oil from being eliminated, potentially giving rise to a food safety problem with even adverse effects on health. 

This indicates that compliance with the labeled content of EPA and DHA is not the only parameter to be investigated when fish oil supplements are analyzed. Rather, an analysis of the overall composition of fatty acids should be performed. The next necessary step of the present study will be a wider campaign of measures, also with the analysis of samples of different types (algae oil, for example), in order to check the extent to which these preliminary conclusions may be extended to the Italian market of omega-3 supplements, namely the commercialization of brands that have not undergone any purification and are available at a lower price. This is an aspect never investigated in other works (value for money).

## Figures and Tables

**Table 1:** Fatty acid composition of the three dietary supplements analyzed (mg/100 mg oil).

Fatty Acids	Supplement No. 1	Supplement No. 2	Supplement No. 3
C12:0 (lauric)	n.d.	n.d.	0.14
C13:0 (tridecanoic)	n.d.	n.d.	0.03
C14:0 (myristic)	0.05	0.13	6.29
C14:1 n-5 (myristoleic)	n.d.	n.d.	0.05
C15:0 (pentadecanoic)	0.01	0.06	0.54
C16:0 (palmitic)	0.16	1.32	13.80
C16:1 n-7 (palmitoleic)	0.11	0.44	7.71
C16:2 n-4 (palmitolinoleic)	n.d.	0.08	1.21
C17:0 (margaric)	0.06	0.10	0.35
C18:0 (stearic)	2.94	4.27	2.77
C18:1 n-9 (oleic)	2.87	6.17	9.55
C18:1 n-7 (vaccenic)	1.29	2.48	2.72
C18:2 n-6 (linoleic)	0.52	1.33	0.99
C19:0 (nonadecanoic)	0.39	0.52	0.37
C18:3 n-3 (α-linolenic or ALA)	0.34	0.70	0.72
C18:4 n-3 (Stearidonic)	0.47	1.54	2.75
C20:0 (arachidic)	0.93	1.02	0.38
C20:1 n-11 (gadoleic)	0.73	0.26	n.d.
C20:1 n-9 (gondoic)	3.58	1.61	0.93
C20:2 n-6 (dihomolinoleic)	0.39	0.13	n.d.
C20:4 n-6 (arachidonic)	2.56	1.59	0.78
C20:4 n-3 (omega-3 arachidonic)	1.58	1.32	0.69
C20:5 n-3 (eicosapentaenoic or EPA)	32.41	25.76	16.68
C22:1 n-11 (cetoleic)	2.60	1.42	n.d.
C22:1 n-9 (erucic)	0.18	0.44	n.d.
C22:5 n-3 (docosapentaenoic or DPA)	2.71	2.91	1.37
C22:6 n-3 (docosahexaenoic or DHA)	19.68	20.06	12.89
EPA + DHA	52.09	45.82	29.57
Σn-3	57.19	52.29	35.10
Σn-6	3.47	3.05	1.77
Σn-6/Σn-3 (dimensionless value)	0.06	0.06	0.05
Σ saturated fatty acids (ΣSFA)	4.54	7.42	24.67
Σ monounsaturated fatty acids (ΣMUFA)	11.36	12.82	20.96
Σ polyunsaturated fatty acids (ΣPUFA)	60.66	55.42	38.08
Other fatty acids	13.58	12.07	8.72
Sum (total FAs)	90.14	87.73	92.43

n.d.: not detected.

**Table 2:** Compliance with the label of dietary supplements analyzed (mg/100 mg oil).

Supplement	EPA			DHA		
	Label	Measured	%	Label	Measured	%
Supplement no. 1	40	32.41	81	20	19.68	98
Supplement no. 2	33	25.76	78	22	20.06	91
Supplement no. 3	15	16.68	111	10	12.89	129

**Table 3:** Analysis of the fish oil test material from fapas^®^.

Fatty Acid	Units	Test Material		Measured Values ^a^	
		Assigned Value	Satisfactory Range	Mean	RPD (%)
18:3 ω-3	%	0.95	0.86–1.05	0.95	6.32
20:5 ω-3	%	12.93	11.64–14.23	11.10	2.97
22:5 ω-3	%	1.88	1.69–2.07	1.74	3.45
22:6 ω-3	%	10.66	9.59–11.73	11.99	7.26

^a^ number of analyses = 2; RPD: relative percent difference.

## Data Availability

The data presented in this study are openly available in Zenodo at DOI:10.5281/zenodo.4425583.

## Sample Availability

Samples of the dietary supplements analyzed are not available from the authors.
